# Intrabronchial application of extracellular histones shows no proinflammatory effects in swine in a translational pilot study

**DOI:** 10.1186/s13104-021-05704-7

**Published:** 2021-07-23

**Authors:** Robert Ruemmler, Alexander Ziebart, Elisabeth Britten, Moritz Gosling, Rene Rissel, Erik K. Hartmann

**Affiliations:** Department of Anesthesiology, Medical Centre of the Johannes Gutenberg University, Langenbeckstrasse 1, 55131 Mainz, Germany

**Keywords:** Extracellular histones, ARDS, Sepsis, Porcine, Lung damage, Experimental

## Abstract

**Objective:**

Extracellular histones have been identified as one molecular factor that can cause and sustain alveolar damage and were linked to high mortality rates in critically ill patients. In this pilot study, we wanted to validate the proinflammatory in vivo effects of local histone application in a prospective translational porcine model. This was combined with the evaluation of an experimental acute lung injury model using intrabronchial lipopolysaccharides, which has been published previously.

**Results:**

The targeted application of histones was successful in all animals. Animals showed decreased oxygenation after instillation, but no differences could be detected between the sham and histone treatments. The histologic analyses and inflammatory responses indicated that there were no differences in tissue damage between the groups.

**Supplementary Information:**

The online version contains supplementary material available at 10.1186/s13104-021-05704-7.

## Introduction

The acute respiratory distress syndrome (ARDS) is a regularly encountered life-threatening condition that often requires treatment in intensive care units and mechanical ventilation [1].

Several rodent [2] and porcine [3] models have been used to identify new therapeutic options and inflammatory pathways to target; the porcine models show the strongest similarity to human physiology and ventilation patterns, thus providing clinically reliable insights into the cause-effect relations of pathophysiologic phenomena. In the past decade, the discovery of the proinflammatory effects of extracellular histones and neutrophil extracellular traps (NETs) has been reported, and their roles in the development of multiorgan damage and ARDS during sepsis have been discovered [4–7]. Most importantly, lung tissue appears to be highly susceptible and vulnerable to elevated histone levels, suggesting a direct correlation between histone serum concentrations and ARDS development [8, 9].

In this pilot study, we tried to translate previously shown proinflammatory effects of histones in rodents [2] to swine to confirm and validate the existing data as well as to evaluate the therapeutic implications in a more clinically accurate setting. Additionally, we designed a standardized acute lung injury model for lipopolysaccharides using the same methods. This report has already been published elsewhere [10]. We hypothesized that overdoses of histones should have comparable effects in swine and would cause tissue inflammation and alveolar leakage in the lung as well as potential septic organ damage.

## Main text

### Materials and methods

#### Animals

Fourteen male German landrace pigs (12–16 weeks, 28–35 kg) were acquired from a local private farm and were treated as described previously [10, 11].

#### Intervention

Following the baseline measurements, flexible bronchoscopy was performed using a single-use fiberoptic bronchoscope (Ambu aScope Regular, Ambu GmbH, Bad Nauheim, Germany). The right and left caudal main bronchi were identified and inspected, and after confirmed insertion of the endoscope into the respective bronchus, the animals were randomized into two groups.

*Group 1 (“Intrabronchial Histones”), n* = *7:* 50 ml of a saline solution containing 100 mg of mixed calf thymus histones (LS002548, Worthington Biochemical Corp, Lakewood, NJ, USA) was instilled through the bronchoscope into each caudal main bronchus separately, adding up to a total of 100 ml and 200 mg of histones.

*Group 2 (“Sham”), n* = *7:* 50 ml of a saline solution without any additives was instilled through the bronchoscope into each caudal main bronchus separately, adding up to a total of 100 ml.

#### Monitoring

After the intervention, the animals were monitored for 8 h, and sample collection was performed as described below. During the monitoring period, the mean arterial blood pressure was kept over 60 mmHg using a norepinephrine drip if necessary and glucose was substituted to maintain the levels above 80 mg/dl. The ventilation parameters were adjusted according to the ARDS network guidelines [12] once oxygen saturation decreased below 93%.

#### Measurements/sample collection

Cardiopulmonary data were constantly measured and collected during the duration of the experiment using a Datex Ohmeda S5 monitor (GE Healthcare, Munich, Germany). These variables included respiratory rate, ventilation pressures, oxygen fractions, oxygen saturation, intra-arterial blood pressure, pulmonary artery pressure, heart rate and core temperature. Additionally, blood gas analyses and cardiac output (CO) measurements were taken at baseline and every hour after the intervention as described before [11].

After termination, both lungs were harvested, and samples from the cranial and caudal left lung lobes (central dorsal and ventral) were either snap frozen for biomolecular analyses or preserved in 2% formaldehyde solution for histologic fixation. Histopathologic scoring and interleukin 6 (IL-6) and tumor necrosis factor alpha (TNFα) expression analyses were performed via ELISA and RT-PCR as described previously [13, 14].

As the primary outcome parameter, pulmonary function represented by the Horovitz ratio (PaO_2_/F_i_O_2_) was determined. Secondary outcomes were the histological organ damages and the proinflammatory cytokine expressions.

The experiment was terminated with the animals being euthanized using high doses of propofol (200 mg) and potassium chloride (40 mmol).

#### Statistical analysis

Since this was a pilot study with no previous data to draw from, no adequate animal number calculation could be performed and animal numbers were chosen empirically. Statistical analyses were performed using 2-way ANOVA intergroup tests with a post hoc Bonferroni correction for repeated measurements as well as Mann–Whitney U test for single measurements via GraphPad Prism 8 software (GraphPad Software, Inc., La Jolla, CA, USA). Data in the text are presented as the mean (standard deviation). P-values < 0.05 were considered significant.

### Results

The interventions were carried out successfully in all animals without alterations to the study protocol. No problems occurred during the endotracheal intubation or while placing the fiberoptic bronchoscope. All 14 animals survived the 8 h monitoring period.

**Fig. 1:**
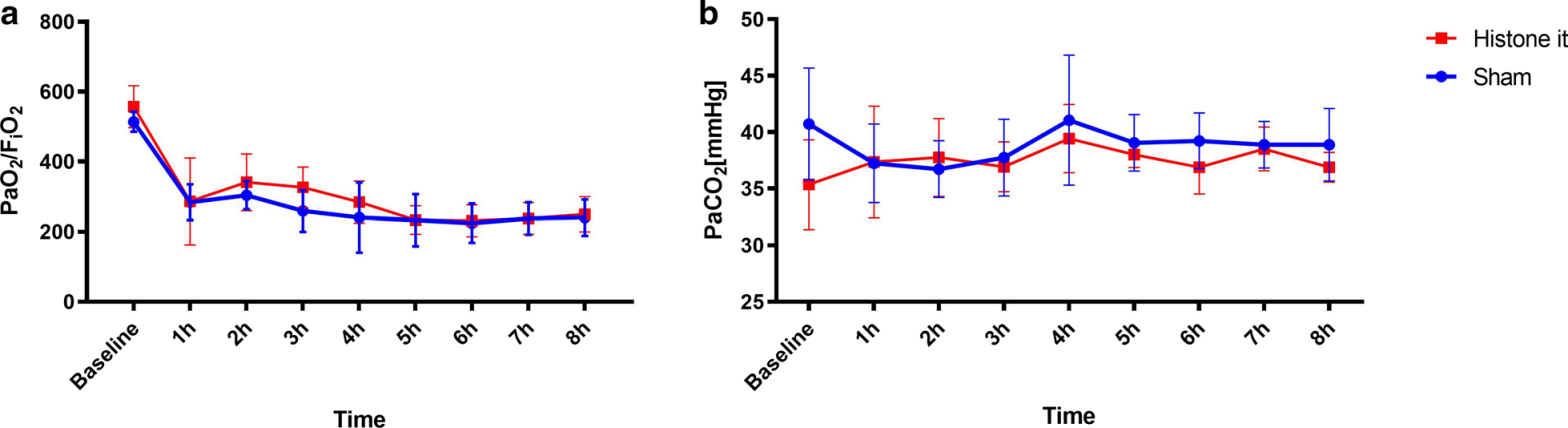
Oxygenation and gas exchange over the 8 h monitoring period. The Horovitz ratio (**a**) decreased significantly after endotracheal fluid instillation but showed no relevant intergroup difference at any time. Decarboxylation (**b**) did not differ significantly

**Fig. 2:**
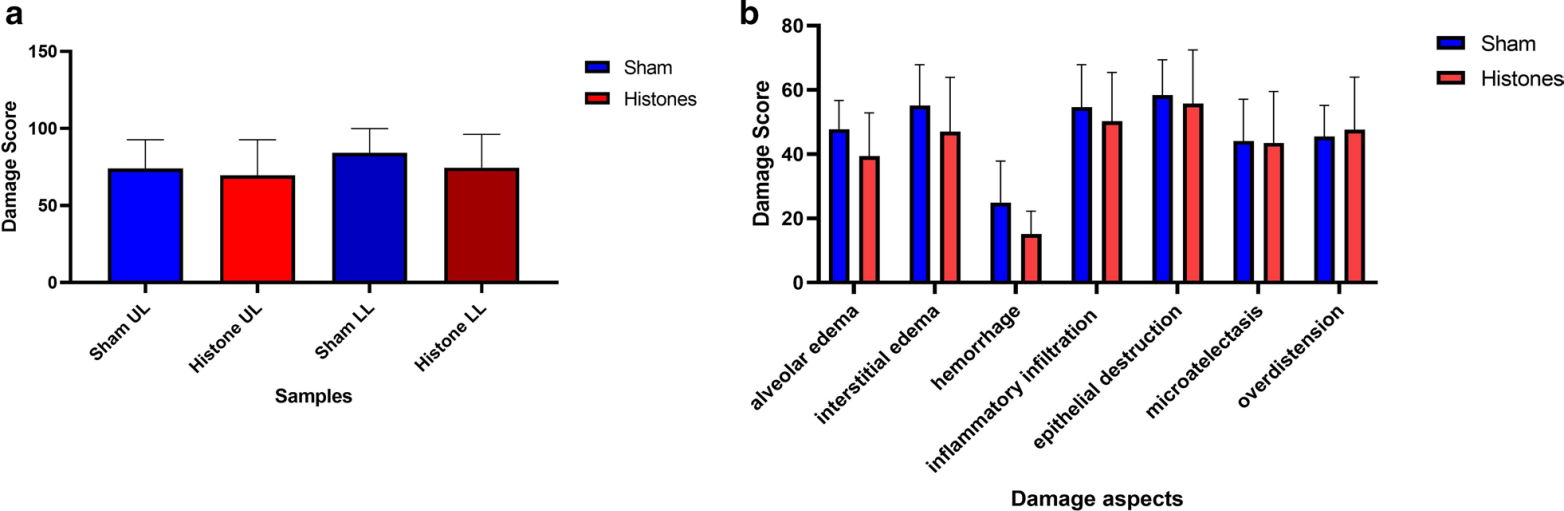
Histologic damage scoring modified after Ziebart et al. [13, 14] evaluating 7 damage aspects (alveolar edema, interstitial edema, hemorrhage, inflammatory infiltration, epithelial destruction, microatelectasis and overdistension. Direct comparisons of samples from the cranial lobe (UL) and caudal lobe (LL) (**A**). Histone-treated animals showed no significant increase in lung damage independent of sample origin. A tendency towards higher scores in the dependent lung areas was not significant and mostly due to intraalveolar edema following instillation. Detailed damage aspect analysis (**B**) of pooled lung tissue samples. No significant shifts are seen for any damage criteria, suggesting the summative score is consistent. The intravenously administered animals were not analyzed since no pulmonary effects were seen

In the intrabronchial instillation groups, there were no significant differences in the vital parameters, including the heart rate (HR), mean arterial blood pressure (MAP), pulmonary pressure (PAP) and central venous pressure (CVP), between the sham and histone treatment groups (Table 1). The oxygenation and gas exchange decreased significantly after instillation but showed no intergroup differences and no significant changes during the monitoring period compared to those of the sham group (Fig. 1). The inspiratory pressures increased accordingly after the intervention, stayed elevated during the monitoring period and did not differ between the instillation groups (see Additional file 1). Neither IL-6 nor TNFα expression was elevated in the lung tissue samples after the histone treatment (see Additional file 1). Histologic damage scoring showed a tendency towards higher tissue damage in the dependent lung areas after instillation but showed no statistical significance and no differences between the interventions (Fig. 2).

**Table 1:** Collected data on haemodynamic parameters, respiratory measurements and blood gas analyses

Parameter Mean (SD)	Baseline	1 h	2 h	4 h	6 h	8 h
HR [bpm]						
Sham	79 (8)	79 (13)	75 (12)	92 (9)	86 (11)	83 (15)
Histone it	74 (18)	79 (28)	71 (16)	91 (24)	88 (22)	85 (21)
MAP [mmHg]						
Sham	71 (5)	84 (7)	84 (7)	80 (7)	75 (8)	75 (12)
Histone it	65 (10)	77 (10)	74 (12)	77 (8)	72 (9)	78 (13)
CVP [mmHg]						
Sham	8 (2)	9 (4)	10 (3)	11 (4)	9 (3)	10 (3)
Histone it	6 (2)	9 (2)	9 (1)	9 (1)	8 (1)	8 (2)
PAP [mmHg]						
Sham	28 (9)	33 (5)*	35 (7)*	33 (6)*	29 (4)*	30 (8)*
Histone it	18 (4)	23 (8)	27 (7)*	28 (6)*	23 (5)	26 (6)
CI [(l/min)/m^2^]						
Sham	3.5 (0.8)	3.5 (0.7)	3.4 (0.3)	3.6 (0.4)	3.9 (0.5)*	3.6 (0.5)*
Histone it	2.9 (0.8)	2.8 (0.4)	3.0 (0.5)	3.5 (0.5)	3.9 (0.5)*	3.8 (0.5)*
NE [mg/h]						
Sham	0	0.06 (0.15)	0	0	0	0
Histone it	0	0.1 (0.19)	0.27 (0.49)	0.14 (0.38)	0.14 (0.38)	0.14 (0.38)
T [°C]						
Sham	36.7 (0.8)	37.5 (0.6)	37.6 (0.4)	37.7 (0.5)	37.9 (0.5)	37.7 (0.2)
Histone it	35.8 (0.7)	36.7 (0.8)	36.8 (0.6)	37.6 (0.6)	37.7 (0.5)	37.7(0.8)
FRC [ml]						
Sham	662 (105)	452 (104)*	428 (103)*	389 (107)*	464 (66)*	496 (44)*
Histone it	772 (197)	501 (158)*	535 (172)*	471 (98)*	463 (107)*	468 (103)*
Lactate [mmol/l]						
Sham	1.8 (1.2)	1.58 (0.49)	1.29 (0.39)	0.79 (0.31)	0.5 (0.09)	0.5 (0.22)
Histone it	1.3 (0.53)	1.56 (0.47)	1.57 (0.61)	0.99 (0.3)	0.67 (0.16)	0.7 (0.31)
SvO_2_ [%]						
Sham	67 (7)	55 (9)	54 (9)	54 (16)	55 (6)	50 (10)
Histone it	67 (8)	50 (13)	53 (13)	60 (10)	56 (9)	52 (9)

HR: heart rate; MAP: mean arterial pressure; CVP: central venous pressure; PAP: pulmonary arterial pressure; NE: norepinephrine; CI: cardiac index; T: temperature; FRC: functional residual capacity; SvO_2_: central venous oxygen saturation; it: intratracheal

### Discussion

This study—for the very first time—tried to apply recent findings regarding the toxicity of extracellular histones in a prospective, randomized fashion in an established intensive care setting in pigs. While there were no relevant technical difficulties in performing the experiments or administering the agents, no significant inflammatory effects could be detected in the intervention groups. Although the intrabronchially instilled animals showed compromised pulmonary function in both groups, no pulmonary inflammation, circulatory failure, fever, or any other pathological effects over the 8 h monitoring period could be detected. While retrospective studies in humans with ARDS, sepsis or acute pancreatitis showed correlations between the histone serum levels and increased mortality rates [2, 6, 15], we did not observe any correlations in the deterioration of vital signs.

Pathological histone concentrations in scientific analyses vary and are estimated to be 5–50 µg/ml [15, 16]. To increase the likelihood of quantifiable results, we used intrabronchial dosages that amounted to approximately 50 µg/ml but still did not detect any effects. Although the intrabronchially instilled animals showed a significant decrease in gas exchange and oxygenation, these results were expected and most likely due to the fluid congesting and compromising the lung. However, no additional impairments were observed beyond the initial drop in PaO_2,_ although previous experiments by our group showed significantly increased inflammation when the same protocol was used with lipopolysaccharide instillation [10]. While histones have been described to be toxic to epithelial and endothelial cells in vitro [2, 5], no specific inflammatory changes were detected in either of the lung damage groups. Additionally, no proinflammatory reactions were detected at the molecular level, suggesting no negative influence even with direct tissue contact of the solution.

### Conclusions

Although non-physiological high doses of histones were used, previously described proinflammatory reactions or organ damage could not be replicated in swine in this experimental setting.

## Limitations

Due to the pilot character and lack of pre-existing data on the use in large animals, the study has several technical and design limitations. First, it can be argued that only nonspecific histone isomer mixtures were used. While the use of purified histone preparations is costly, the isolation and production of single isomer agents is even more sophisticated and expensive, especially when considering their use in large animals. However, studies have suggested that histone toxicity may vary between isomers and post-translational modifications and affect different organs depending on the isoform they are exposed to [17, 18], thus potentially affecting the observable effects. Additionally, while bovine histone preparations do affect murine cell lines, there is no data on their effects on porcine tissue. Notably, the studies that revealed potentially increased mortality rates due to high amounts of circulating histones in critically ill patients only used nonspecific screening assays for determination of the histone concentrations in patient serum samples [15, 16]. Additionally, most cell culture and rodent experiments use histone mixtures of at least 4–6 isomers [2, 5, 19]. The histone preparation used in this trial was a mixture of calf thymus histones produced in comparatively large batches. While there is no preliminary data on proinflammatory effects of this exact preparation, the quality and purity of the proteins is guaranteed by the manufacturer and the agent has been used to identify histone interactions before [20]. Given the fact, that various different histone preparations have been used in the trials cited in this manuscript and have been shown to induce inflammation in different tissues, the expectation of respective effects of this histone solution can be warranted. Since intrabronchial instillation has never been performed in pigs, loss of agents either due to chemical characteristics, adhesion to the endoscope or even an unexpected species-specific increased resistance to histone effects cannot be sufficiently excluded. To quantify this problem, we would need to perform exact serum concentration analyses as well as a dose–response titration trial or porcine cell inoculations, which were not carried out in this study due to the pilot character and infrastructural limitations. This is a major flaw, since the quality of the used solution could not be verified prior to its use.

Second, the instillation method could be criticized as flawed with a high potential of confounding factors due to relatively high instillation volumes and significantly decreased pulmonary function in both the sham and histone groups, most likely due to intra-alveolar edema and atelectasis. However, in another trial of our group, the exact same method was used with comparable initial declines in PaO_2_ after instillation of 20 mg lipopolysaccharide (LPS), and we could still detect significant increases in tissue inflammation as well as septic circulatory effects [10]. LPS and histones should affect similar pathways, heavily relying on Toll-like receptors 2 and 4 [2, 4]. Hence, a comparable inflammatory response, especially to high doses considering the amounts used and measured in cell cultures and intensive care patients, could reasonably be expected. Additionally, the targeted administration method via bronchoscopy allows for direct control of the drug application and should prevent unnecessary losses caused by prolonged airway passage compared to blind intratracheal injection or other untargeted methods [21–23].

Third, a large animal model for analysis of the histone effects and intensive care treatment is needed. While the data mentioned above clearly indicate the toxic potential of histones in vitro, the in vivo data are solely based on retrospective sample collection or correlation analyses with no randomized prospective approach. Additionally, the finding that the detected histone concentrations in critically ill patients with the worst outcomes tend to be the highest allows no deduction of direct causation, since nonspecific histone detection could also stem from substantial cell death, which is expected in patients suffering from multiorgan failure. This hypothesis is supported by the fact that the most impressive human clinical data on the serum concentrations of histones stem from trauma and pancreatitis patients [15, 24], with both entities being linked to extensive tissue damage and necrosis; these factors potentially explain the amount of liberated nuclear proteins without direct evidence of inflammatory mediation through histones. However, most groups supported their findings with in vitro testing and showed cytotoxic effects on organ tissue-derived cell lines [2, 25, 26]. Since we did not have direct access to murine models or cell culture experiments during this study, we could not confirm the toxicity of our prepared solution.

## Supplementary Information

**Additional file 1.** Proinflammatory marker assessment and ventilation data.

## Data Availability

All data generated or analysed during this study are included in this published article.

## Abbreviations

ARDS: Acute respiratory distress syndrome
CO: Cardiac output
CVP: Central venous pressure
ELISA: Enzyme-linked immunosorbent assay
FRC: Functional residual capacity
HR: Heart rate
LPS: Lipopolysaccharide
MAP: Mean arterial pressure
NET: Neutrophil extracellular traps
PAP: Mean pulmonary artery pressure
RT-PCR: Real-time polymerase chain reaction
