# Supplementary material for: Intrabronchial application of extracellular histones shows no proinflammatory effects in swine in a translational pilot study
Source: BMC Res Notes. 2021 Jul 23;14:285. doi: 10.1186/s13104-021-05704-7 (PMC8306385; doi:10.1186/s13104-021-05704-7)
Supplement: Supplementary file 1 — Additional file 1. Proinflammatory marker assessment and ventilation data. [file 13104_2021_5704_MOESM1_ESM.docx]

**Additional Files supporting Manuscript “Intrabronchial Application of Extracellular Histones Shows no Proinflammatory Effects in Swine in a Translational Pilot Study”**

**
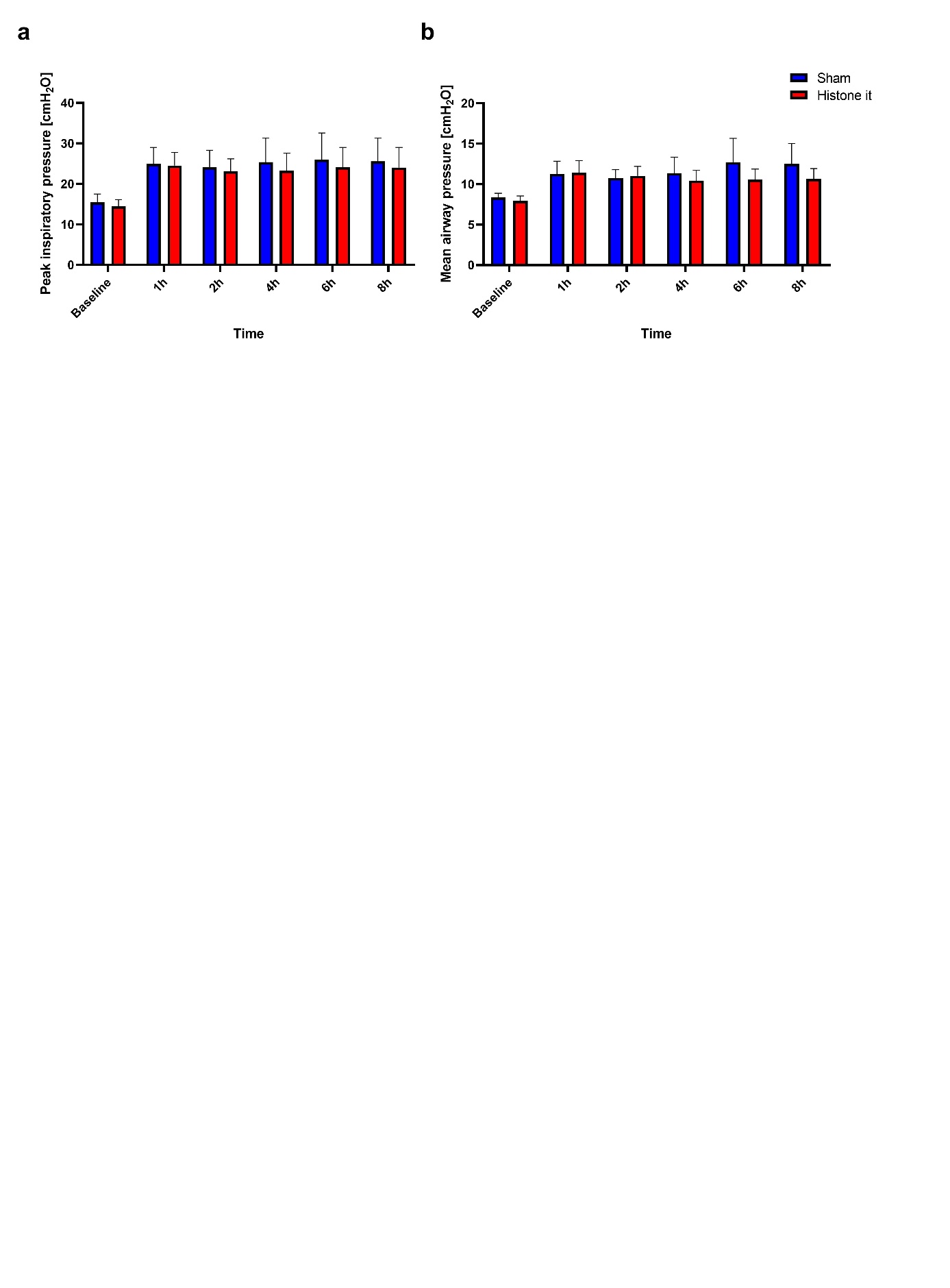
**

**Figure S1:** Inspiratory pressures over an 8 h monitoring period of sham and histone tracheal instillation groups. Peak inspiratory pressure (a) and mean airway pressure (b) were significantly increased (P<0.01) after instillation and remained elevated in both groups without showing relevant intergroup differences. The intravenously administered animals showed no changes to baseline and no other respiratory symptoms (data not shown).

**
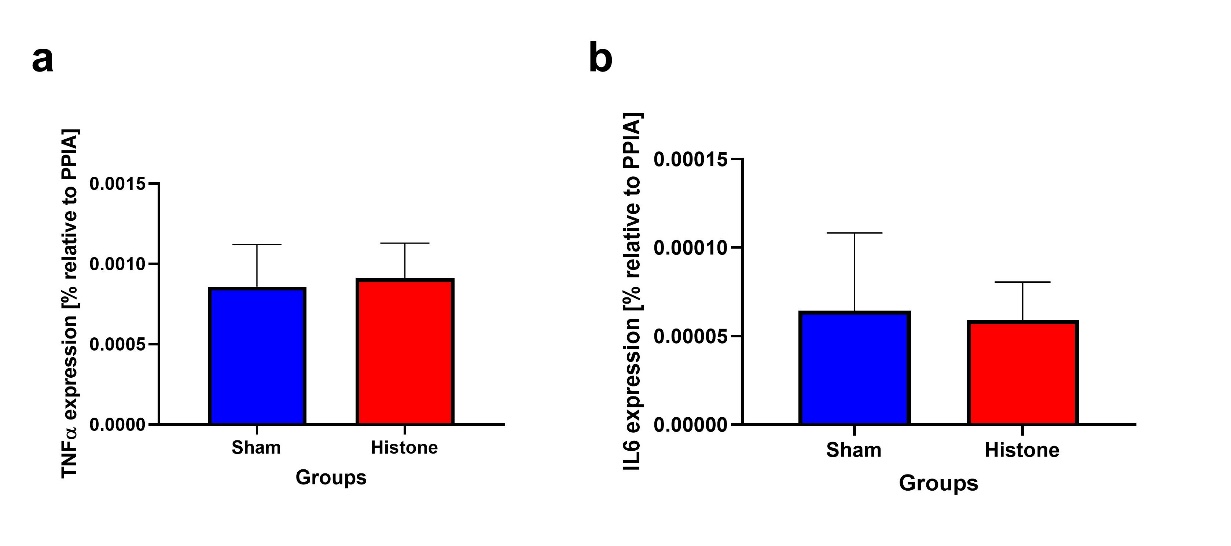
**

**Figure S2:** Inflammatory marker expression of TNF-alpha (a) and Interleukin-6 (b) in the lung tissues relative to Peptidylprolyisomerase A (PPIA) . No significant differences could be detected between the groups. Intravenous groups were not analyzed due to the lack of clinical indication for inflammation.
